# Analysis and Optimization of Dimensional Accuracy and Porosity of High Impact Polystyrene Material Printed by FDM Process: PSO, JAYA, Rao, and Bald Eagle Search Algorithms

**DOI:** 10.3390/ma14237479

**Published:** 2021-12-06

**Authors:** Manjunath Patel Gowdru Chandrashekarappa, Ganesh Ravi Chate, Vineeth Parashivamurthy, Balakrishnamurthy Sachin Kumar, Mohd Amaan Najeeb Bandukwala, Annan Kaisar, Khaled Giasin, Danil Yurievich Pimenov, Szymon Wojciechowski

**Affiliations:** 1 Department of Mechanical Engineering, PES Institute of Technology and Management, Visvesvaraya Technological University, Belagavi 590018, India; 2 Department of Mechanical Engineering, KLS Gogte Institute of Technology, Visvesvaraya Technological University, Belagavi 590018, India; ganeshchate@gmail.com (G.R.C.); Amaan.b@gmail.com (M.A.N.B.); annankaisar@gmail.com (A.K.); 3 Department of Mechanical Engineering, B.M.S. College of Engineering, Visvesvaraya Technological University, Belagavi 590018, India; vineeth.p2000@gmail.com (V.P.); sachinkumarb.mech@bmsce.ac.in (B.S.K.); 4 School of Mechanical and Design Engineering, University of Portsmouth, Portsmouth PO1 3DJ, UK; Khaled.giasin@port.ac.uk; 5 Department of Automated Mechanical Engineering, South Ural State University, Lenin Prosp. 76, 454080 Chelyabinsk, Russia; danil_u@rambler.ru; 6 Faculty of Mechanical Engineering and Management, Poznan University of Technology, 60-965 Poznan, Poland

**Keywords:** JAYA, high impact polystyrene, bald eagle search, fused deposition modelling, particle swarm optimization, cylindricity error, porosity

## Abstract

High impact polystyrene (HIPS) material is widely used for low-strength structural applications. To ensure proper function, dimensional accuracy and porosity are at the forefront of industrial relevance. The dimensional accuracy cylindricity error (CE) and porosity of printed parts are influenced mainly by the control variables (layer thickness, shell thickness, infill density, print speed of the fused deposition modeling (FDM) process). In this study, a central composite design (CCD) matrix was used to perform experiments and analyze the complete insight information of the process (control variables influence on CE and porosity of FDM parts). Shell thickness for CE and infill density for porosity were identified as the most significant factors. Layer thickness interaction with shell thickness, infill density (except for CE), and print speed were found to be significant for both outputs. The interaction factors, i.e., shell thickness and infill density, were insignificant (negligible effect) for both outputs. The models developed produced a better fit for regression with an R^2^ equal to 94.56% for CE, and 99.10% for porosity, respectively. Four algorithms (bald eagle search optimization (BES), particle swarm optimization (PSO), RAO-3, and JAYA) were applied to determine optimal FDM conditions while examining six case studies (sets of weights assigned for porosity and CE) focused on minimizing both CE and porosity. BES and RAO-3 algorithms determined optimal conditions (layer thickness: 0.22 mm; shell thickness: 2 mm; infill density: 100%; print speed: 30 mm/s) at a reduced computation time equal to 0.007 s, differing from JAYA and PSO, which resulted in an experimental CE of 0.1215 mm and 2.5% of porosity in printed parts. Consequently, BES and RAO-3 algorithms are efficient tools for the optimization of FDM parts.

## 1. Introduction

Effective waste management in fabricating parts to desired shapes at low cost led to the development of 3-D printing technology, also called additive manufacturing (AM) [1]. Industry 4.0 also aims at developing extremely high material, efficiency-based AM processing routes for industrial-scale production of parts [2]. To date, the 3D printing technique is popular for fabricating simple or custom-designed parts on a small scale [3]. Mould preparations are essential to fabricate parts using conventional manufacturing (say, injection molds), which alone account for several thousand dollars [4]. However, AM techniques do not require high-cost molds to fabricate parts. AM techniques are thereby applied to fabricate parts useful for aerospace, civil, biomedical, surgical implants, automobiles, electronics, and so on [5,6]. This has led to rapid progress in the global market with an estimated increased rate of ≈17% compound annual growth rate [3]. 

AM techniques are classified according to the state of starting materials, i.e., liquid (stereolithography, multi-jet modeling), filament/paste, freeze-form extrusion fabrication (FDM), powder (selective laser sintering, selective laser melting, electron beam melting, laser metal deposition (LMD), 3-dimensional printing), and solid sheet laminated object manufacturing). All techniques differ in the way they build layers [4,5,6,7]. Rapid progress in AM techniques is primarily due to their ability to reduce assembly costs, as they quickly print the complex geometry parts from CAD files by depositing two-dimensional layers on a building platform [8]. FDM is the preferred processing technique for modeling, prototyping, and production due to its significant advantages over other AM routes such as (a) reduced maintenance cost for fabricating complex geometries without tooling requirements, (b) ease of operation without demanding experts or human interface, (c) a wide range of material fabrication without exposure to toxic chemicals, and (d) fast processing routes [1,9,10]. The said benefits of the FDM process in fabricating functional parts do have shortcomings such as poor surface integrity and mechanical properties, low accuracies, and so on [8]. To satisfy customers and increase FDM market share, the product quality must be improved regarding better surface integrity, dimensional accuracy, and production cost. Determining the optimal set of parametric conditions needs to be established [11]. In recent years, worldwide attention has been paid to determining the right variables to attain the desirable properties in fused deposition modeling parts [12,13]. Process efficiency and parts quality are affected by a large number (say, more than 15) of intervention process variables related to the fused deposition modeling process [8,11,13]. Studying many intervention variables experimentally with varying one-factor-at-a-time approaches is impractical, expensive, and time-consuming [14]. Statistical experimental design and artificial intelligence tools showed improved performance of the one-factor-at-a-time approach [15]. 

In recent years, experimental design methods (Taguchi, response surface methodology [RSM]) were employed to perform experiments and analyze variables influencing FDM parts quality [16,17,18,19,20,21,22,23]. The Taguchi method, helpful in a preliminary screening of parameters concerning a large number of variables, although the existence of complex non-linear relationships and interaction effects of factors are not estimated [16,17,19,20,22,23]. Interaction factors must not be neglected when providing detailed insight into a process [24]. Interaction factors are important because there is a difference in factor effects to produce the same effect on the output quality characteristics at different levels corresponding to other process variables [24,25]. It was proven that considering all terms (main factors and their interactions) resulted in drawing precise process information and offered better predictions than neglecting interaction factors and insignificant factor effects [24,25,26]. RSM methods limit the said shortcomings by estimating the effects of individual factors, curvature effects, and their interaction could offer precise information with better process prediction capability [24]. ABS is the material most used to fabricate parts [16,17], followed by PLA [27], nylon [23], and ASA [22] viz. FDM process. Layer thickness is the most significant parameter for both BT and SR of ABS parts [16], whereas the infill variable percentage showed the highest contribution towards the mechanical properties of PLA parts [27]. LT contributions concern more the length thickness of ASA parts [22]. Speed showed a significant impact on reducing feedstock material consumption and build time, with higher dynamic flexural modulus on ABS parts [28]. High impact polystyrene (HIPS — polybutadiene added to polystyrene during polymerization), categorized as a low-strength structural application material, is widely used for the pre-production of prototypes due to their availability, low cost, ease of fabrication, machining, paint, and glue [29,30]. HIPS is used as a potential substitute for sand in cement mortar due to its technological benefits (mortar becomes ductile, increases energy dissipation, decreases the bulk density, etc.) [31]. The surface and mechanical properties of HIPS were improved when subjected to the electroplating technique [32]. Interlayer strengths of parts processed viz. material extrusion resulted in a lack of strong interlayer bonding due to poor diffusion [33]. Note that an appropriate choice of variables could improve the material properties. Furthermore, little research is being done on high-impact polystyrene material and study process variables that could yield better quality in FDM parts.

Table 1 shows that the contribution of each factor varies when they are analyzed with different variables on multiple outputs. The desirability function approach (DFA) was used to optimize the multiple outputs simultaneously [22,28]. Artificial intelligence tools outperformed the desirability function approach in determining optimized conditions of different manufacturing processes [14,25,34,35,36]. This could be due to the typical characteristics and search mechanisms of different algorithms [14]. The RSM method reveals maximum information (establishing relationships mathematically between process variables and parts quality) regarding the process with limited experimental trials or runs [18,28,37]. Note that algorithms (GA-NN, GA-ANFIS) produced better-optimized process conditions than RSM predictions [18,37]. This occurred due to search mechanisms carried out at many spatial locations in a multi-dimensional search space [14]. Table 1 also shows that the level range considered for the parameters (layer thickness, for example) differed for the same materials [16,18]. From the literature review, RSM proved an efficient technique to study process variables offering better process insights and product quality. 

In product assembly, cylindrical features (dimension properties) are important quality characteristics that ensure the proper functioning of parts [38,39]. Cylindricity errors are treated as areas between two co-axial cylinders, among which all points of the part surface must lie [40]. JAYA, PSO, GA, and TLBO have been applied to simultaneously optimize the dimensional errors (surface roughness, cylindricity error, circularity error) in turning and drilling processes [38,39]. GA was applied to conduct a global search to minimize the deviation of building parts compared with STL facets of 3D geometry [41]. Note that the density of the PETG based on FDM parts strongly affects the mechanical properties [42]. Note that meta-heuristic algorithms (GA, PSO, DE, BFOA, SOS, NSGA-II) require tuning algorithm-specific parameters, which not only increases the computational complexity but also affects the solution accuracy [21,43,44]. Precise tuning of algorithm-specific parameters is difficult and a tedious task for practice engineers and researchers. Recently, Rao proposed three metaphor less algorithms that optimize the processor to determine solutions with simple mathematical steps [45]. PSO was applied to minimize the dimensional deviations (i.e., circularity and flatness error) caused by the FDM process [46]. RSM was applied to model the process, followed by multi-objective optimization by applying PSO. JAYA algorithm showed good accuracy for 24 benchmark problems. [47]. Bald eagle search (BES) optimization outperformed many algorithms in terms of producing better solution accuracy, due to the exhaustive search mechanisms considering the best features of the swarm and evolutionary algorithms [48]. Rao algorithms (RAO-1, RAO-2, and RAO-3) outperformed other meta-heuristic algorithms for solving optimization problems [45]. Note that little research effort was applied to BES, PSO, JAYA, and Rao-algorithms that could focus on minimizing the cylindricity errors and maximizing the density of FDM parts.

HIPS material has proven its potential in developing prototypes, tooling for food industries, low strength molding, optoelectronic, chemical, and biological applications [29,30,31,49]. The applications can be enhanced by notably improving the internal (reduced porosity) and external features (improved dimensional accuracy) of printed parts. In the present work, the systematic methodology was conducted to minimize the geometrical deviations (cylindricity error) and maximize the density of HIPS parts. Porosity was treated as an output parameter as it affected the mechanical properties, whereas geometrical deviation in assembly parts resulted in malfunction during operation. Experiments were conducted to analyze the parameters that influence part-quality characteristics (cylindricity error and density) using RSM. BES, PSO, JAYA, and Rao-algorithms (RAO-3) were applied to predict the optimized conditions (layer thickness, shell thickness, infill density, speed) of fused deposition modeling parts. The performance of algorithms was tested for both solution accuracy and computation time. The predicted optimal conditions were validated by conducting confirmation experiments. 

## 2. Materials and Methods

High Impact Polystyrene (HIPS) material is widely applied for developing prototypes and low-strength structural applications due to its economic and technical benefits [29,30]. The properties of HIPS material are presented in Table 2. 

**Figure 1:**
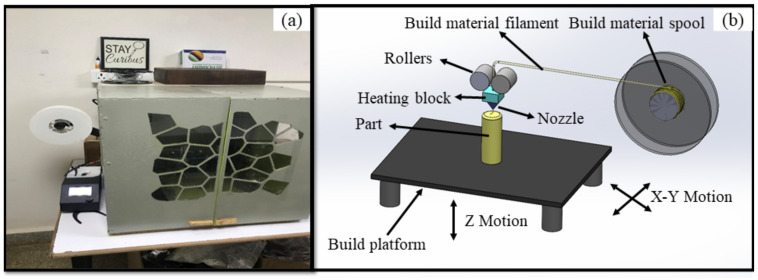
Experimental set-up: (**a**) CR 10 3D printer, and (**b**) schematic view of FDM printer.

The FDM process uses wire (thermoplastic) drawn from a spool and fed through the nozzle wherein the rollers set the direction for deposition. The material to be deposited is heated initially to a malleable state, and then the material is extruded through nozzles that build the parts. The parts are printed initially bead-by-bead followed by layer-by-layer to fabricate 2-dimensional layers one over the other, in the pre-defined direction or scan path. The material extruded to build parts is allowed to cool and solidify. The thermocouple estimates the nozzle temperature, wherein the temperature of the material to be deposited can be accurately controlled. After ensuring complete deposition of material as per dimensions, which is of the 3-dimensional physical part, it is allowed to cool at room temperature. The printed parts are removed from the machine without damage. Post-processing operations are carried out to remove support structures (if any) irrespective of geometry, size, and fabrication direction, and to improve acceptable surface integrity of parts. To ensure better dimension accuracy (i.e., cylindricity error) and fabricate defect-free parts (i.e., reduced porosity), the appropriate choice of fused deposition modeling parameters is controlled. The experimental set-up used for building parts is presented in Figure 1. 

The set of variables and levels of experimentation were decided on after performing the pilot experiments and referring to the literature presented in Table 1. Input variables and operating levels include such things as layer thickness (0.16, 0.22, 0.28 mm), shell thickness (2, 3, 4 mm), infill density (20, 60, 100%), and print speed (30, 50, 70 mm/sec). 

**Figure 2:**
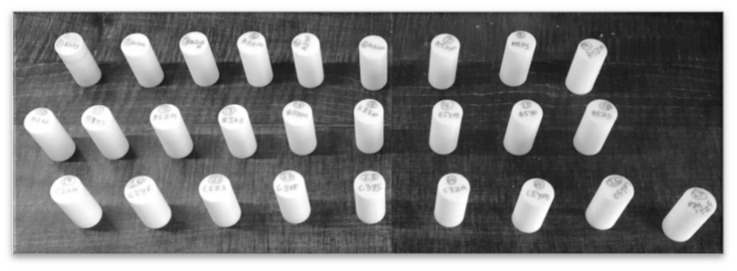
3D printed fused deposition modelling parts.

**Figure 3:**
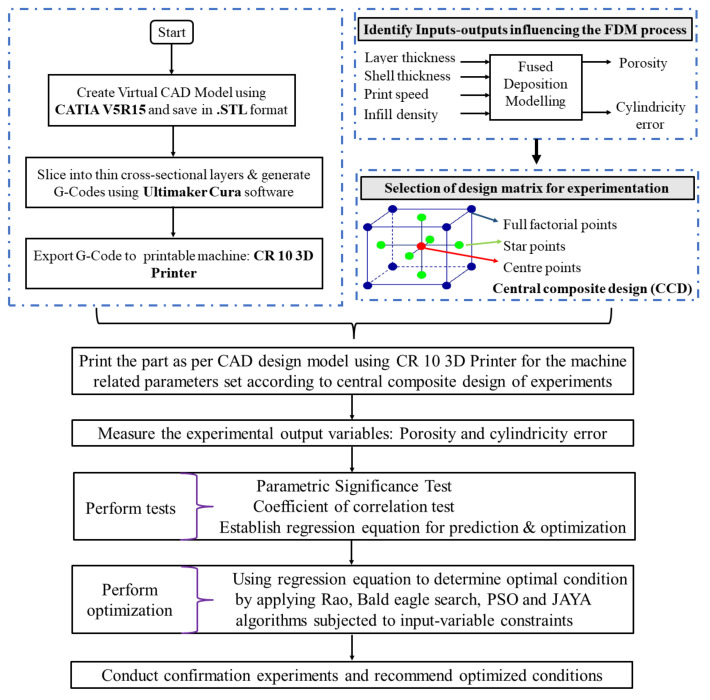
The framework of proposed research work on modelling and optimization.

A CCD-based experimental matrix was used to conduct experiments. An experimental plan representing different sets of fused deposition modeling variables is presented in Table 3. Two parts (replicate) were printed for each set of the experimental trial. A COMET L3D Tripod column type 3D scanner measured the cylindricity error of print samples. Archimedes’ principle was used to measure the porosity of FDM parts. The average values of two replication experiments on the printed sample were used to collect the output (cylindricity error and porosity) data. RSM was applied to perform statistical analysis (factor effects of individual, quadratic, and interaction) that analyzed input parameter outputs. Furthermore, RSM developed regression equations (input-output) for conducting predictions and optimization. A few of the printed samples are presented in Figure 2. The framework proposed for modeling, analysis, and optimization of the fused deposition modeling process is presented in Figure 3.

## 3. Results and Discussions

The experimental input/output data collected per the face-centered central composite design is presented in Table 3. The average values of two replication experiments on the printed sample were used to collect the output (cylindricity error and porosity) data (refer to Table 3). The maximum and minimal error from mean values of response data (experimental data) of all 27 experiments (presented in Table 3) were found equal to +0.58% and −0.87% for porosity, +0.03 mm, and −0.05 mm for cylindricity error. The response-wise analysis was performed to determine detailed insight regarding the influence of input variables.

### 3.1. Response: Cylindricity Error

The mathematical equation relating the cylindricity error (CE) and input variables derived from the experimental data was presented in Equation (1).

(1) $$\begin{array}{l} \mathrm{CE}\;=0.868\;-6.08\;A\;+0.204\;B\;+0.00519\;C\;-0.0220\;D\;+29.71\;A^{2}+0.1224\;B^{2}\;\\ \;-0.000071\;C^{2}\;\;\;+0.000141\;D^{2}-3.453\;\mathrm{AB}\;-0.0051\;\mathrm{AC}\;+0.0534\;\mathrm{AD}\;\\ \;+0.000932\;\mathrm{BC}\;-0.00228\;\mathrm{BD}\;\;\;+0.000081\;\mathrm{CD} \end{array}$$

**Figure 4:**
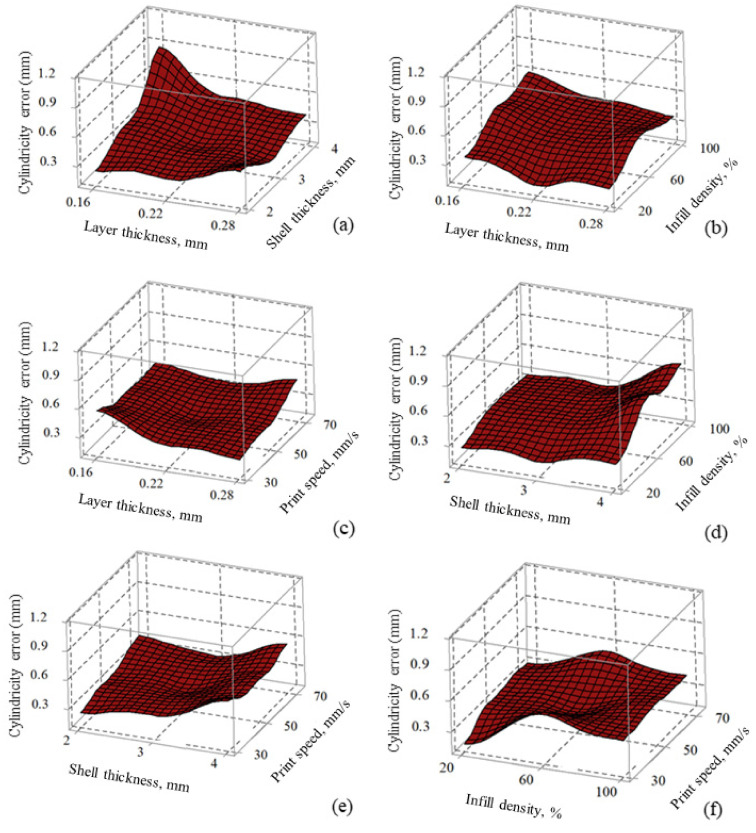
Surface plots of cylindricity error vs. (**a**) LT and ST, (**b**) LT and ID, (**c**) LT and PS, (**d**) ST and ID, (**e**) ST and PS, and (**f**) ID and PS.

Figure 4a shows the printing factors’ effects on cylindricity error. An increase in layer thickness (from 0.16–0.28 mm) and shell thickness (2–4 mm) increased the cylindricity error (refer to Figure 4a). The combined increase in layer thickness and shell thickness increased the material mass, causing gravity force and induced forces influencing the deformation to be higher and generating geometric (i.e., cylindricity) error. Low values of layer thickness (number of discrete points on the edge) imply light-cured resin due to layer-by-layer deposition at no definite axis or plane [50]. The thin layer created more discrete points, ensuring an accurate print by producing smooth, uniform, and detailed features [51]. Although printing time decreased with increased layer thickness [52], the thicker layer generated few discrete points and separated from themselves at extended distances, leading to a stair-stepping effect at the edge affecting the dimensional accuracy [50].

Parts built with a lower shell thickness (2–3 mm) showed lower cylindricity error. Lower shell thickness resulted in higher dimension accuracy with better product economy, as observed in the literature [53]. The combination of low values of layer thickness (0.16 mm) and high values of shell thickness (4 mm) showed higher cylindricity error (refer to Figure 4a). Low values of infill density (20%) resulted in lower cylindricity error in fabricated parts (refer to Figure 4b,d,f), analogous to earlier results reported by authors [54]. Lower infill density (quantity of material in the part) used less material. Therefore, heat loss due to solidification process, from higher to room temperature without generating significant thermal stresses, causing no significant variations in part dimensions [18]. Higher infill density (100%), although recommended to get high strength parts, needs to compensate with increased material consumption, printing time, and associated high cost [55]. The influence of printing speed (the rate at which melted material is extruded and deposited) was found insignificant (because the resulting surface plots seemed to be almost flat when varied between their respective levels) for cylindricity error (refer to Figure 4c,e–f). Although lower print speed (30 mm/s) may not be recommendable as it is not economical, high speed may have a greater possibility of missing melt deposition at the desired location, resulting in dimensional variations (cylindricity errors) on solidified surfaces [55]. An increase in print speed (30–70 mm/s) tends to increase the variation in the volume of material deposited by the toolpath, causing dimensional instability (variations in the material deposited between Intra and interlayer elements) in printed parts. 

### 3.2. Response: Porosity

The second-order polynomial response equations representing porosity as a function of input variables are presented in Equation (2).

(2) $$\begin{array}{l} \mathrm{Porosity}\;=17.21-70.8\;A\;+0.954\;B\;-0.1085\;C\;-0.0663\;D\;-6.5\;A^{2}-0.4205\;B^{2}\\ \;-0.000042\;C^{2}\;-0.000335\;D^{2}+11.57\;\mathrm{AB}\;+0.1963\;\mathrm{AC}\;+0.7326\;\mathrm{AD}\;\\ \;+0.00282\;\mathrm{BC}\;-0.02599\;\mathrm{BD}\;\;\;+0.000436\;\mathrm{CD}\; \end{array}$$

**Figure 5:**
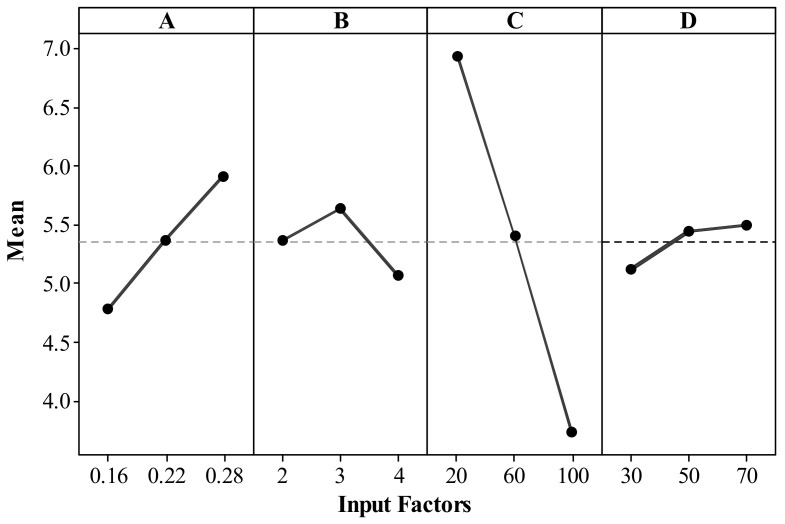
Main effect plots for porosity.

Figure 5 shows individual factor effects on the porosity of FDM parts. An increase in layer thickness (from 0.16–0.28 mm) resulted in an increased percentage of porosity in FDM parts. An increased number of layers introduces voids between the layers, due to differences in density at bonding interfaces and the complex nature of thermoplastic polymer which is viscoelastic and viscoplastic in behavior [56]. A similar trend was observed in fiber-reinforced thermoplastic composites [57]. Figure 5 showed high values of shell thickness (4 mm), desirable to yield less porosity in the fused deposition modeling parts, which might occur due to lack of diagonal filling with too small a shell thickness (2 mm). An appropriate choice of wall or shell thickness does not allow internal infill and thereby significantly improves the impermeability of the product [58]. Increased infill density (from 20–100%, introduced to create a porous structure in the parts which tends to reduce the weight) resulted in developing stronger parts (i.e., low porosity), as shown in Figure 5. Smaller pores are observed with higher infill density (100%), which increases the strength of the parts, as reported in the literature [59]. It was observed that print speed (velocity at which the print head moves during printing) does not show a significant impact on the porosity of fused deposition modeling parts (refer to Figure 5). This occurs due to the vibrations and errors introduced during the printing process at higher print speed (70 mm/s) led to increased porosity in parts [60]. 

### 3.3. Analysis of Variance of Responses: Cylindricity Error and Porosity

To statistically examine the models developed for cylindricity error and porosity, the analysis of variance tests was performed. 

Table 4 shows the combined effects of all linear factors (layer thickness, shell thickness, infill density, print speed). Their square and 2-term interactions are found to have significant (i.e., *p*-value ≤ 0.05) for both outputs. Note that the statistical significance of the factors was tested for the pre-defined confidence level of 95%. All linear terms (except print speed for cylindricity error) are found significant for both the outputs. The impact of shell thickness and infill density is comparatively higher for cylindricity error, unlike the layer thickness and infill density for porosity on the printed parts. The impact of print speed is negligibly small for both cylindricity error and porosity (refer to Figure 4c,e–f, and Figure 5). Although print speed effects are insignificant, their interaction with layer thickness and infill density for cylindricity error is significant. Noteworthy is that print speed contributions with interactions among the layer thickness, infill density, and shell thickness are significant for porosity. Note that shell thickness interaction with layer thickness is insignificant for both outputs. This indicates the inclusion of non-contributory, i.e., 2-term interaction terms in regression equations, do not change the porosity and cylindricity errors (refer to Table 4 and Equations (1) and (2)). However, excluding non-contributary terms could reduce the prediction precision of a process. The square terms, i.e., print speed, are found to be insignificant (corresponding *p*-value > 0.05), which practically signifies that the relationship between cylindricity error and porosity is linear (refer to Figure 4 and Figure 5, and Table 4). Note that *p*-values of square term correspond to layer thickness, shell thickness, and infill density of less than 0.05, indicating their relationship with cylindricity error is non-linear (refer to Table 4). R^2^ value examines both model accuracies and the goodness of fit of regression. It is important to note that both models showed an R^2^ value close to 100% (i.e., 94.56% for cylindricity error and 99.1% for porosity). This strongly signifies the model is statistically significant for practical utility in industries for predictions and optimization. 

### 3.4. Multi-Objective Optimization Algorithms

#### 3.4.1. Rao Algorithm

Optimizing the conflicting process outputs (to simultaneously optimize for maximizing and minimizing the outputs corresponding to the problem domain) is difficult for industry engineers and assumed to be tedious due to mathematical complexity [43], although many algorithms based on metaphor (mimic behavior of animals, birds, fish, lion, ant, and so on) were applied to solve such problems [61]. However, many algorithms are dying (no takers), perhaps due to the following reasons [45,47,61]: (a) requires solving complex mathematical equations, (b) tuning of algorithm-specific parameters, (c) higher computation time, (d) failure to reproduce optimal global results, (e) inefficiency, (f) expert’s requirement, and so on. In recent years, Rao et al. introduced the new metaphor less and algorithm-specific parameter-less algorithms (Rao Algorithms: RAO-1, RAO-2, and RAO-3) to overcome the above shortcomings [45]. Rao algorithms identify worst and best solutions in the entire population through random interactions (n population, k = 1, 2,…n) during an optimal search at m iterations corresponding to decision variables (d) [43]. For any optimization problem, the fitness function (f) needs to be either maximized or minimized. The fitness function with the best and worst from *n* populations are represented as fbest and fworst, respectively. The value of $X_{k,l,m}$ (i.e., the value of k^th^ variable corresponds to l^th^ candidate at m iteration) is updated according to Equation (3),

(3) $$X_{k,\;l,\;m}^{l}=X_{k,\;l,\;m}+rand_{1,\;k,\;l,\;m}\left(\underset{\begin{array}{c} best\;candidate\;for\\ \;\mathrm{var}iable\;l\;at\;m\;iteration \end{array}}{\underbrace{X_{best,\;l,\;m}}}-\underset{\begin{array}{l} worst\;candidate\;for\\ \mathrm{var}iable\;l\;at\;m\;iteration \end{array}}{\underbrace{X_{worst,\;l,\;m}}}\right)$$

(4) $$\begin{array}{l} X_{k,\;l,\;m}^{l}=X_{k,\;l,\;m}+rand_{1,\;k,\;l,\;m}\left(X_{best,\;l,\;m}-X_{worst,\;l,\;m}\right)\\ \;+rand_{2,\;k,\;l,\;m}\left(\left|X_{k,\;l,\;m}\;\mathrm{or}\;X_{K,\;l,\;m}\right|-\left|X_{K,\;l,\;m}\;\mathrm{or}\;X_{k,\;l,\;m}\right|\right) \end{array}$$

(5) $$\begin{array}{l} X_{k,\;l,\;m}^{l}=X_{k,\;l,\;m}+rand_{1,\;k,\;l,\;m}\left(X_{best,\;l,\;m}-X_{worst,\;l,\;m}\right)\\ \;+rand_{2,\;k,\;l,\;m}\left(\left|X_{k,\;l,\;m}\;\mathrm{or}\;X_{K,\;l,\;m}\right|-\left(X_{K,\;l,\;m}\;\mathrm{or}\;X_{k,\;l,\;m}\right)\right) \end{array}$$

Terms rand_1_ and rand_2_ are random numbers that operate in the range of 0 and 1. Equations (4) and (5), $X_{k,\;l,\;m}\;\mathrm{or}\;X_{K,\;l,\;m}$ represent the k^th^ candidate solution compared with the random K^th^ candidate solution and exchange information corresponding to fitness value. If fitness function f_k_ produced a better function value than f_K_ then $X_{k,\;l,\;m}\;\mathrm{or}\;X_{K,\;l,\;m}$ turns out to be $X_{k,\;l,\;m}$ and the term $X_{K,\;l,\;m}\;\mathrm{or}\;X_{k,\;l,\;m}$ turns out to be $\;X_{K,\;l,\;m}$. Conversely, if the fitness function value of K^th^ candidate solutions produced a better solution, then the fitness function of the k^th^ candidate solution $X_{k,\;l,\;m}\;\mathrm{or}\;X_{K,\;l,\;m}$ turns out to be $X_{K,\;l,\;m}$ and $X_{K,\;l,\;m}\;\mathrm{or}\;X_{k,\;l,\;m}$ turns out to $X_{k,\;l,\;m}$. To attain the optimal global solutions, Equation (3) is used for the RAO-1 algorithm, whereas Equation (4) is for the RAO-2 algorithm and Equation (5) is for the RAO-3 algorithm. The performance of globally optimal solutions of RAO algorithms is compared among themselves after comparing the fitness values, several function evaluations, and time. The RAO-3 algorithm was used to determine optimal conditions for the FDM process.

#### 3.4.2. BES Algorithm

The bald eagle search algorithm combines the desirable features of swarm intelligence (to locate the best position in the swarm) and evolutionary (expand search space to avoid local minima solutions) algorithm [48]. The BES algorithm is a recently introduced algorithm that mimics the intelligent social behavior of bald eagles in locating the best position for a food source (fish) [62]. The BES algorithm demonstrated good accuracy for benchmark problems and hence was chosen for this problem [48]. Bald eagles search for food sources in three stages [48,61]: 

Stage 1 Selecting space: identify the area that could ensure bald eagles locate the food source space using Equation (6).

(6) $$P_{new,\;i}=P_{best}+\left[\alpha \times r\left(P_{mean}-P_{i}\right)\right]$$

The term r can be any random value between 0 and 1. P_best_ is the previous best position of bald eagles in the search space. α is the parameter whose role is to control the changes in position and the corresponding value maintained between 1.5 to 2. The α value maintained is equal to 1.5. P_new_ corresponds to the new position of bald eagles. P_mean_ depicts the eagles using up all information from the previous points.

Stage 2 searching space: eagle initiates a search for a food source (prey) from the selected search space in spiral shape using Equation (7).

(7) $$P_{i,new}=P_{i}+\left[\frac{yr\left(i\right)}{\max\left|yr\right|}\times \left(\;P_{i}-P_{i+1}\right)\right]+\left[\frac{xr\left(i\right)}{max\left|xr\right|}\times r\left(P_{i}-P_{mean}\right)\right]\;$$

The term, $xr\left(i\right)=r\left(i\right)\times sin(\theta \left(i\right)$), $yr\left(i\right)=r\left(i\right)\times cos\left(\theta \left(i\right)\right)$, $\theta \left(i\right)=\alpha \times \pi \times rand$ and $r\left(i\right)=\theta \left(i\right)\times R\times rand$.

Term, a parameter determines the corner between point search in the central point whose value lies between 5 and 10, and in the present work, the value of a is kept equal to 10. R depicts the search cycles and the values lie between 0.5 and 2. In the present work, R is kept fixed equal to 1.5. 

Stage 3 swooping: eagle starts with the best point defined in the search space and carryout further movements to attack prey. Solutions are identified based on the best solution in a swooping manner using Equation (8).

(8) $$P_{i,\;new}=rand\;*\;P_{best}+\left[\frac{xr\left(i\right)}{max\left|xr\right|}\times \left(P_{i}-c_{1}\times P_{mean}\right)\right]+\left[\frac{yr\left(i\right)}{\max\left|yr\right|}\times \left(P_{i}-c_{2}\times P_{best}\right)\right]$$

The term, $xr\left(i\right)=r\left(i\right)\times \sinh(\theta \left(i\right)$), $yr\left(i\right)=r\left(i\right)\times \cosh\left(\theta \left(i\right)\right)$, $\theta \left(i\right)=\alpha \times \pi \times rand,\;r\left(i\right)=\theta \left(i\right)$.

In Equation (8), C_1_ and C_2_ is the eagle movement towards the best and centre point, and those values are maintained equal to 2. After ensuring the optimal search is concluded, the point corresponding to the minimum value of the objective function is chosen as the local best only when it produced a lower value than the previous best. 

#### 3.4.3. JAYA Algorithm

The JAYA algorithm development is credited to Rao [47], which is an algorithm-specific parameter-less (does not require tuning of algorithm parameters) algorithm. Note that the JAYA algorithm also requires tuning population size and iterations. JAYA algorithm outperformed TLBO, GA, and DE in determining optimal solutions tested against 24 benchmarking problems [47]. The search mechanisms to determine solutions for the problem domain are done based on the concept of moving toward the best solution while simultaneously avoiding the worst. The solutions corresponding to best and worst are determined through the defined size of the population. The new solutions are determined by considering the best and worst solutions according to Equation (9).

(9) $$\begin{array}{l} \underset{new\;solution}{\underbrace{X\prime_{j,k,i}}}=\\ X_{j,k,i}+\underset{\begin{array}{c} random\;number\\ \left[0,1\right] \end{array}}{\underbrace{rand_{{}_{1,j,i}}}}\;\left(\underset{best\;solution}{\underbrace{X_{j,best,i}}}-│X_{j,k,i}│\right)-\underset{\begin{array}{c} random\;number\\ \left[0,1\right] \end{array}}{\underbrace{rand_{2,j,i}}}\;\left(\underset{worst\;solution}{\underbrace{X_{j,worst,i}}}-│X_{j,k,i}│\right) \end{array}$$

The term j is the decision variable (for the present work, 4), k and i represent the candidate in the population at iterations i. $X_{j,k,i}$ value represents the j^th^ decision variable corresponding to the k^th^ candidate at i^th^ iteration. The new solutions determined viz. $X\prime_{j,k,i}$ are compared with $X_{j,k,I}$ and the better solution of the two is updated. This procedure is carried out for pre-defined iterations and populations till it ensures optimal solutions are determined.

#### 3.4.4. PSO Algorithm

Swarm intelligence-based PSO is well known worldwide to optimize various manufacturing domains [25,36,63]. PSO mimics the swarm behavior of fish or birds in nature to guide particles towards global solutions (search for food) [64]. In PSO, swarm refers to a group of particles, wherein each particle is initialized randomly and all fly in multi-dimensional search space in search for food. The objective functions are then evaluated to conclude optimal solutions for pre-defined iterations. In PSO, each particle moves with a certain velocity and adjusts its flight path in accordance with experience gained through self-flying (cognitive leader, Personal best: P_s_) and neighbor (social leader, Global best: P_g_) particles. In each iteration, the P_s_ and P_g_ of particle velocity and positions are determined and updated using Equation (10).

(10) $$\vec{V}\left(i+1\right)=w\times \vec{V}\left(i\right)+\left[\vec{P}s\left(i\right)-\vec{P}\left(i\right)]+[\vec{P}g\left(i\right)-\vec{P}\left(i\right)\right]$$

(11) $$\vec{P}\left(i+1\right)=\vec{P}\left(i\right)+\vec{V}\left(i+1\right)\;\;$$

where w is the inertia weight, $V\left(i\right)$ is the original velocity of the particle, $\vec{V}\left(i+1\right)$ is the updated velocity of the particle. $P_{s}\left(i\right)-P\left(i\right)$ term depicts the relative direction between swarm best and present position of the particle and $P_{g}\left(i\right)-P\left(i\right)$ is the relative direction between the global best and present position of the particle. The positions of particles are updated according to Equation (11). 

### 3.5. Results of Optimization Models

The regression equations derived from experimental data were subjected to application optimization techniques (BES, PSO, JAYA, and RAO-3) that could reduce both cylindricity error and porosity of the 3D printed samples. The performance of all 4 algorithms is compared in terms of solution accuracy and computation time. 

#### 3.5.1. Mathematical Formulation for Multi-Objective Optimization

The present work comprises two objectives that require optimization for obtaining minimum cylindricity error and porosity value. The mathematical regression equations established viz. design of experiments were treated as objective functions for performing optimization tasks (refer to Equations (1) and (2)). In the present work, two objectives required minimized values, and hence it became a multi-objective problem. To solve a multi-objective problem, a single equation needed to be established which accounts for the optimization of both porosity and cylindrical error using Equation (12).

(12) $$\min f(z)=\left[w_{1}\times \frac{porosity}{porosity_{min}\;}\;\right]+\left[w_{2}\times \frac{cylindricity\;error}{cylindricity\;error_{min}\;}\;\right]\;$$

The goal was to minimize the *f*(z) by altering decision variables layer thickness A: 0.16–0.28 mm; shell thickness B: 2–4 mm; Infill density C: 20–100%; and print speed D: 30–70 mm/s. Terms *w*_1_ and *w*_2_ were weights that corresponded to porosity and cylindricity error. Terms $porosity_{min}$ and cylindricity $error_{min}$ were the minimum values that corresponded to porosity and cylindricity error. A single objective optimization task was carried out by all four algorithms to determine $porosity_{min}$ and $Cylindricity\;error_{\min}$. All four algorithms (BES, PSO, JAYA, and RAO-3) were coded on Python (3.8.0) and executed on a computer (HP Intel (R) Core (TM) i3-7100U CPU at 2.40 GHz and RAM: 4G) to minimize *f*(z) and thereby minimize cylindricity error and porosity.

#### 3.5.2. Estimating Solution Accuracy and Determining Optimal Conditions

All four algorithms determined values of $porosity_{min}$= 0.904% for a set of variables, i.e., the layer thickness of 0.17 mm, shell thickness of 2 mm, infill density of 20%, print speed of 56.31 mm/s, and $Cylindricity\;error_{\min}$ = 0.0659 mm corresponded to a layer thickness of 0.16 mm, shell thickness of 4 mm, infill density of 100%, and print speed of 70 mm/s. 

It was observed that $porosity_{min}$ was obtained for print speed of 56.31 mm/s, keeping layer thickness, shell thickness, and infill density at minimal value. Conversely, $Cylindricity\;error_{\min}$ was obtained when shell thickness, infill density, and print speed were maintained at high values, whereas layer thickness was maintained at a minimal value. Although the nature of optimization for both outputs is minimization, input variables conflicted with one another. The solution accuracy differed (optimal input condition that minimized porosity may not minimize cylindricity error and vice versa) for the conflicting input behavior on outputs. Therefore, weight factors were assigned for individual outputs (*w*_1_ and *w*_2_ are weight factors for porosity and cylindricity error), respectively. Six cases were considered, giving equal weight (case 1) importance to both outputs (*w*_1,_ and *w*_2_ = 0.5) and maximum importance (case 2–6) to one output minimal to the rest. Note that the summation of weight factors (*w*_1,_ + *w*_2_ = 1) must be maintained equal to 1. The objective functions were evaluated to determine the fitness function value (solving Equation (12)) corresponding to different case studies (different sets of weights) by applying four algorithms. Note that all algorithms are capable of producing approximately similar results, and the obtained results are presented in Table 5. It was observed that the fitness function values differed from one another due to the different weight fractions (importance given to individual output) assigned to the individual output. The objective functions defined to minimize the fitness function value (goal to minimize both cylindricity error and porosity), and therefore, case 4 (porosity; *w*_1 =_ 0.4, and cylindricity error *w*_2_ = 0.6) were recommended as optimal fused deposition modeling conditions due to their lower fitness function value equal to 2.494. Table 5 presents the results of optimal conditions corresponding to the FDM process subjected to different case studies. 

#### 3.5.3. Estimate Computation Time and Solution Accuracy in Determining Optimal Conditions

Examining the computation time when generating the optimal solutions is of industrial relevance (example: reduce product development time in the automotive industry) [65]. The computation time varies for different algorithms based on algorithm-specific parameters and search mechanisms determining optimal global solutions. The goal of any optimization problem is to attain higher solution accuracy at reduced computation efforts and time. The optimal solution and the corresponding decision variable values should be well-established for attaining good quality manufacturing parts. In general, if the number of iteration and population size decreases below the threshold value, the solution might converge to local minima rather than global minima. Therefore, all four (i.e., PSO, RAO-3, JAYA, BES) algorithms are executed for 1000 iterations along with a population size of 50. All four algorithms converge on the same input condition, which establishes the solution accuracy or global minimum (refer to Table 5). 

To obtain the least possible value of computational time, both population and the number of iterations are to be minimized. Each algorithm has a different value of iterations and population below which accuracy is compromised. To validate models in terms of computation efficiency, all four algorithms are executed with common iterations and population size equal to 100 and 20 for Trial 1, and 300 and 10 for Trial 2, respectively. RAO-3 and BES algorithms converge at the same fitness value, i.e., 2.546 tested for case 1 (refer to Table 5) and computation time (refer to Table 6). For Trial 1 and Trial 2 conditions, JAYA and PSO algorithms converge to a sub-optimal solution (i.e., close to global optima). Although JAYA didn’t converge at the global minima, the error was small and executed in 0.011 s. Therefore, the JAYA and PSO algorithms require more iterations and population size to attain the global fitness function value. Table 6 shows that the BES and RAO-3 algorithms predicted optimal conditions requiring less computation time to attain optimal global solutions. That the PSO algorithm requires more computation time for both trials than RAO-3, BES, and JAYA algorithm might be due to algorithm search mechanisms, tuning of algorithm-specific parameters, and so on.

#### 3.5.4. Confirmation Experiments

**Figure 6:**
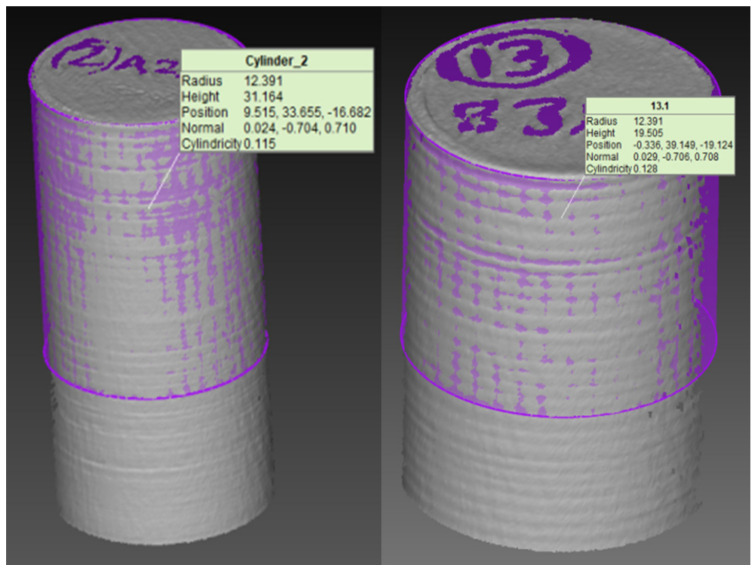
Cylindricity error was obtained for optimized conditions.

Experiments were conducted in optimized conditions (Case 4: layer thickness is 0.22 mm, shell thickness is 2 mm, infill density is 100%, print speed is 30 mm/s) to validate the optimization models. Case 4 (assigning 40% importance to porosity, and 60% importance to cylindricity error) was recommended by all algorithms as the optimal condition for FDM parts. Two replicates were prepared for the optimized fused deposition modeling condition. The resulting average values of cylindricity error and porosity of printed parts were found equal to 0.1215 mm, and 2.5%. A few optimized condition samples resembling the cylindricity error are presented in Figure 6. The algorithm’s predictions and experimental data for optimized conditions were closely mapped with one another and resulted in quality parts (minimized values of porosity and cylindricity error). It can be concluded that RSM is a useful tool for modeling and statistical analysis that delivers detailed process insights. In addition, RSM-derived equations are useful to determine optimal conditions through search algorithms. The successful BES and RAO-3 algorithms can certainly be utilized for performing optimization tasks for different domains of manufacturing problems.

## 4. Conclusions

The minimum values of cylindricity error and porosity in FDM parts are indeed essential for proper functioning during operations in many applications. The results of the experimental study, analysis, and optimization of the FDM process is presented below.

1. All factors (except print speed for CE) were found statistically significant for both outputs. Shell thickness was the major contributing factor for cylindricity error, whereas least significant for the porosity of printed samples. Infill density was the most significant factor for porosity.
2. The print speed relationship with cylindricity error and porosity was found to be linear, whereas shell thickness was found to have a non-linear relationship.
3. All the interaction factor effects were significant, except the interactions among shell thickness and infill density (for CE and porosity) and layer thickness and infill density (for CE). Insignificant terms practically imply a lesser contribution to the outputs of a process. Both models produced better fit with a value of 99.1% for porosity and 94.56% for cylindricity error, respectively.
4. Four algorithms (BES, RAO-3, PSO, and JAYA) were applied to determine the optimal fused deposition modeling conditions. Six case studies (set of weight fractions assigned to both outputs) were analyzed and the optimal conditions were determined. Case 4 (layer thickness 0.22 mm, shell thickness 2 mm, infill density 100%, print speed 30 mm/s) is recommended as the optimal condition, as they produced a minimum fitness value equal to 2.494. The recommended optimal conditions are experimentally evaluated and the resulting cylindricity error and porosity of printed parts were found equal to 0.1215 mm, and 2.5%.
5. The computational time of all four algorithms (BES, RAO-3, PSO, and JAYA) were tested with common iterations and population size. BES and RAO algorithms were converged (population size: 20; iterations: 100) to optimize global solutions with a computation time equal to 0.007 s. JAYA and PSO algorithms converge on local solutions for population size: 20; iterations: 100, and require more population size and iteration to attain global solutions.
6. BES and Rao algorithms are computationally efficient for attaining global solutions and efficient tools for optimizing FDM parts.

## Figures and Tables

**Table 1:** Summary of literature review of FDM process parameters and their optimization.

Materials	Experimental Method	Process Variables	Analyzed Parameters	Remarks	Ref.
	Optimization Method				
ABS	Taguchi method	LT: 0.254–0.3302 mm; ID: SHD-SLD; SST: Sparse, smart	BT, SR	LT showed the highest contributions for both BT and SR. SLD and smart support style produce better results for both BT and SR.	[16]
ABS	Taguchi method	LT: 0.16–0.24 mm; CT: 35–55 °C; ET: 207–230 °C; PT: 110–132 °C; NS: 1–3; IDM: 0.8–1.2; ISS: 0.56–0.84; FT: 0.64–0.94 mm; IP: H, L, D; ID: 25–75%; IS: 72–108 mm/s; OS: 24–40 mm/s; ISS: 54–90 mm/s.	DA	The set of high values of IS, IP, mid-values of CT, LT, PT, NS, IDM, FT-linear, ISS, and low values of OS, ID, ISSM, and ET resulted in better dimensional accuracy of parts.	[17]
ABS	RSM method	LT: 0.12–0.4 mm; BO: 0–90°; ID: 0–100%; NC: 2–10	DA	ANN-GA predictions and optimization results are better than RSM-GA.	[18]
	RSM-GA & ANN-GA				
PLA	Taguchi method	LT: 0.1–0.3 mm; PS: 70–110 mm/s; NT: 220–240 °C; filling style: raster (short, long and offset); RW: 0.3–0.5 mm.	Distortion	Fast filling speed, low nozzle temperature, and layer thickness offset raster style ensures smaller distortion	[20]
PLA	RSM method	ID: 20–100%; T: 190–210 °C; PS: 50–150 mm/s	TS	↑ID and T, with mid-values of speed results in ↑TS. GA-ANN produced better results than other methods.	[37]
	GA-RSM, GA-ANN, GA-ANFIS				
PLA	RSM method	LT: 0.18–0.3 mm; PS: 36–60 mm/s; PT: 185–205 °C; OSS: 29–40 mm/s	SR	PS and LT showed significant contributions to SR. PSO and SOS predicted identical optimal conditions	[21]
	PSO and SOS				
ASA	Taguchi method	LT: 0.18–0.33 mm; FP: solid, sparse, and hexagonal; BO: 0–90°; PP: XY, XZ, YZ; TP: 1–9	Processing time, EC, width, length, thickness	PP is the most significant factor for ↓process time and EC. FP influences the more on width. LT contributions are more for length thickness. PP influences more on part thickness.	[22]
	DFA				
Nylon	Taguchi method	LT: 0.1–0.3 mm; IFD: 50–100%; PS: 60–70 mm/s	UTS, impact strength, hardness, FS	IFD showed the highest contribution on all outputs. ↓LT is better for all outputs except hardness.	[23]

**Table 2:** Properties of HIPS Material.

Property	Value
Density	1.08 g/cm^3^
Surface Hardness	RM30
Tensile Strength	42 MPa

**Table 3:** Experimental input-output data of the FDM process (CCD).

Input Variables				Output Variables	
Layer Thickness, (mm)	Shell Thickness, (mm)	Infill Density, (%)	Print Speed, (mm/s)	Porosity, (%)	Cylindricity Error, (mm)
0.16	2	20	30	8.17	0.172
0.16	2	60	50	5.64	0.159
0.16	2	100	70	3.21	0.400
0.16	3	20	50	7.36	0.332
0.16	3	60	70	4.46	0.438
0.16	3	100	30	3.27	0.470
0.16	4	20	70	3.81	0.599
0.16	4	60	30	4.98	1.076
0.16	4	100	50	2.15	0.920
0.22	2	20	70	7.63	0.202
0.22	2	60	30	4.41	0.259
0.22	2	100	50	3.87	0.349
0.22	3	20	30	7.35	0.145
0.22	3	60	50	5.71	0.352
0.22	3	100	70	4.50	0.390
0.22	4	20	50	6.63	0.223
0.22	4	60	70	4.99	0.582
0.22	4	100	30	3.27	0.558
0.28	2	20	50	6.30	0.418
0.28	2	60	70	6.85	0.723
0.28	2	100	30	2.26	0.296
0.28	3	20	70	7.98	0.246
0.28	3	60	30	5.17	0.390
0.28	3	100	50	4.95	0.204
0.28	4	20	30	7.22	0.183
0.28	4	60	50	6.42	0.407
0.28	4	100	70	6.11	0.612

**Table 4:** Analysis of variance for cylindricity error & porosity.

Response		Cylindricity Error			Porosity		
Source	DF	Adj. SS	*p*-Value	Significance	Adj. SS	*p*-Value	Significance
Model	14	1.2983	0.000	S	78.074	0.000	S
Linear	4	0.5097	0.000	S	53.145	0.000	S
Layer thickness	1	0.0656	0.007	S	5.7949	0.000	S
Shell thickness	1	0.2645	0.000	S	0.4213	0.020	S
Infill density	1	0.1566	0.000	S	46.271	0.000	S
Print speed	1	0.0229	0.079	IS	0.6576	0.006	S
Square	4	0.2556	0.001	S	1.1985	0.012	S
Layer thickness^2^	1	0.0686	0.006	S	0.0033	0.817	IS
Shell thickness^2^	1	0.0899	0.003	S	1.0608	0.001	S
Infill density^2^	1	0.0781	0.004	S	0.0269	0.512	IS
Print speed^2^	1	0.0190	0.106	IS	0.1075	0.201	IS
2-Term Interaction	6	0.5329	0.000	S	23.730	0.000	S
Layer thickness × Shell thickness	1	0.3863	0.000	S	04.338	0.000	S
Layer thickness × Infill density	1	0.0014	0.647	IS	1.9970	0.000	S
Layer thickness × Print speed	1	0.0370	0.031	S	6.9556	0.000	S
Shell thickness × Infill density	1	0.0125	0.182	IS	0.1147	0.188	IS
Shell thickness × Print speed	1	0.0188	0.108	IS	2.4309	0.000	S
Infill density × Print speed	1	0.0374	0.030	S	1.0936	0.001	S
Error	12	0.0746			0.7062		
Total	26	1.3729			78.7802		
		R^2^: 94.56%; R^2^ adjusted: 88.22%			R^2^: 99.10%; R^2^ adjusted: 98.06%		

S: Significant (*p*-value ≤ 0.05); IS: Insignificant (*p*-value > 0.05); DF: degrees of freedom; R^2^: Coefficient of determination; *p*-value: preset confidence value.

**Table 5:** Summary of results of the optimal fused deposition modeling process.

Case Study (*w*1 and *w*2)	Layer Thickness (mm)	Shell Thickness (mm)	Infill Density (%)	Print Speed (mm/s)	Porosity (%)	Cylindricity Error (mm)	Min *f*(z)
Case 1 (*w*_1_, *w*_2_ = 0.5)	0.21	2	100	30	2.62	0.147	2.564
Case 2 (*w*_1 =_ 0.6, *w*_2_ = 0.4)	0.207	2	100	30	2.65	0.145	2.639
Case 3 (*w*_1 =_ 0.7, *w*_2_ = 0.3)	0.18	2.23	20	58.26	2.87	0.15	2.905
Case 4 (*w*_1 =_ 0.4, *w*_2_ = 0.6)	0.216	2	100	30	2.55	0.15	2.494
Case 5 (*w*_1 =_ 0.3, *w*_2_ = 0.7)	0.22	2	100	30	2.49	0.16	2.526
Case 6 (*w*_1 =_ 0.2, *w*_2_ = 0.8)	0.24	2	100	30	2.31	0.20	2.939

**Table 6:** Summary of results of the optimal fused deposition modeling process.

Optimizing Algorithm	Trials (Iterations & Population Size)	Layer Thickness (mm)	Shell Thickness	Infill Density (%)	Print Speed (mm/s)	Computational Time (s)
PSO	Trial 1 (100 & 20)	0.21	2	100	20	0.014
JAYA		0.28	2.5	100	30	0.013
RAO-3		0.21	2	100	30	0.007
BES		0.21	2	100	30	0.007
PSO	Trial 2 (300 & 10)	0.21	2	100	20	0.017
JAYA		0.18	2	100	31	0.013
RAO-3		0.21	2	100	30	0.011
BES		0.21	2	100	30	0.011

## Data Availability

Data can be made available upon request.

## Nomenclature

Porosity _min_	Minimum values of porosity
Cylindricity error _min_	Minimum values of cylindricity error
*f*(z)	Fitness function
$w_{1}$	Weight importance of porosity
$w_{2}$	Weight importance of cylindricity error
w	Inertia Weight
R^2^	Coefficient of correlation
R^2^ Adj.	Adjusted Coefficient of Correlation
A	Layer Thickness
ABS	Acrylonitrile Butadiene Styrene
AM	Additive Manufacturing
ANN	Artificial Neural network
ANFIS	Adaptive Network Fuzzy Interface System
ASA	Amino-Salicylic Acid
B	Shell Thickness
BES	Bald Eagle Search Optimization
BO	Build Orientation
BT	Build Time
C	Infill Density
CAD	Computer Aided Design
CCD	Central Composite Design
CE	Cylindricity Error
CT	Chamber Temperature
D	Print Speed
DA	Dimensional Accuracy
DE	Differential Evolution
DFA	Desirability Function Approach
EC	Energy Consumption
ET	Extruder Temperature
FDM	Fused Deposition Modelling
FP	Filling Pattern
FS	Flexural Strength
FT	Floor Thickness
GA	Genetic Algorithm
HIP	High Impact Polystyrene
IDM	Inset Distance Multiplier
IFD or ID	Infill Density
IP	Infill Pattern
IS	Infill Speed
ISS	Inset Speed
LT	Layer Thickness
NC	Number Of Contours
NS	Number Of Shells
NT	Nozzle Temperature
OS	Outline Speed
OSS	Outer Shell Speed
PC-ABS	Polycarbonate ABS
PLA	Polylactic Acid
PP	Printing Plane
PS	Print Speed
PT	Platform Temperature
PSO	Particle Swarm Optimization
RA	Raster Angle
RSM	Response Surface Methodology
RW	Road Width
SHD	Sparse High Density
SLD	Sparse Low Density
SOS	Symbiotic Organism Search
SR	Surface Roughness
ST	Shell Thickness
SST	Support Style
TS	Tensile Strength
UTS	Ultimate Tensile Strength

## References

[1] Attaran M. (2017). The rise of 3-D printing: The advantages of additive manufacturing over traditional manufacturing. Bus. Horiz..

[2] Mehrpouya M., Dehghanghadikolaei A., Fotovvati B., Vosooghnia A., Emamian S.S., Gisario A. (2019). The potential of additive manufacturing in the smart factory industrial 4.0: A review. Appl. Sci..

[3] Lee C.H., Padzil F.N.B.M., Lee S.H., Ainun Z.M.A.A., Abdullah L.C. (2021). Potential for Natural Fiber Reinforcement in PLA Polymer Filaments for Fused Deposition Modeling (FDM) Additive Manufacturing: A Review. Polymers.

[4] Kamara S., Faggiani K.S. (2021). Fundamentals of Additive Manufacturing for the Practitioner.

[5] Bhargav A., Sanjairaj V., Rosa V., Feng L.W., Fuh Y.H.J. (2018). Applications of additive manufacturing in dentistry: A review. J. Biomed. Mater. Res. Part B Appl. Biomater..

[6] Haleem A., Javaid M. (2019). Additive manufacturing applications in industry 4.0: A review. J. Ind. Inf. Integr..

[7] Prakash K.S., Nancharaih T., Rao V.S. (2018). Additive manufacturing techniques in manufacturing-an overview. Mater. Today Proc..

[8] Dey A., Yodo N. (2019). A systematic survey of FDM process parameter optimization and their influence on part characteristics. J. Manuf. Mater. Process..

[9] Lalegani Dezaki M., Mohd Ariffin M.K.A. (2020). The effects of combined infill patterns on mechanical properties in FDM process. Polymers.

[10] Konta A.A., García-Piña M., Serrano D.R. (2017). Personalised 3D printed medicines: Which techniques and polymers are more successful?. Bioengineering.

[11] Mohamed O.A., Masood S.H., Bhowmik J.L. (2015). Optimization of fused deposition modeling process parameters: A review of current research and future prospects. Adv. Manuf..

[12] Rajpurohit S.R., Dave H.K. (2018). Effect of process parameters on tensile strength of FDM printed PLA part. Rapid Prototyp. J..

[13] Popescu D., Zapciu A., Amza C., Baciu F., Marinescu R. (2018). FDM process parameters influence over the mechanical properties of polymer specimens: A review. Polym. Test..

[14] Patel G.C.M., Chate G.R., Parappagoudar M.B., Gupta K. (2020). Machining of Hard Materials: A Comprehensive Approach to Experimentation, Modeling and Optimization.

[15] Nor N.M., Mohamed M.S., Loh T.C., Foo H.L., Rahim R.A., Tan J.S., Mohamad R. (2017). Comparative analyses on medium optimization using one-factor-at-a-time, response surface methodology, and artificial neural network for lysine–methionine biosynthesis by *Pediococcus pentosaceus* RF-1. Biotechnol. Biotechnol. Equip..

[16] Wankhede V., Jagetiya D., Joshi A., Chaudhari R. (2020). Experimental investigation of FDM process parameters using Taguchi analysis. Mater. Today Proc..

[17] Mahmood S., Qureshi A.J., Talamona D. (2018). Taguchi based process optimization for dimension and tolerance control for fused deposition modelling. Addit. Manuf..

[18] Deswal S., Narang R., Chhabra D. (2019). Modeling and parametric optimization of FDM 3D printing process using hybrid techniques for enhancing dimensional preciseness. Int. J. Interact. Des. Manuf..

[19] Chohan J.S., Kumar R., Singh T.B., Singh S., Sharma S., Singh J., Mia M., Pimenov D.Y., Chattopadhyaya S., Dwivedi S.P. (2020). Taguchi S/N and TOPSIS Based Optimization of Fused Deposition Modelling and Vapor Finishing Process for Manufacturing of ABS Plastic Parts. Materials.

[20] Xinhua L., Shengpeng L., Zhou L., Xianhua Z., Xiaohu C., Zhongbin W. (2015). An investigation on distortion of PLA thin-plate part in the FDM process. Int. J. Adv. Manuf..

[21] Saad M.S., Nor A.M., Baharudin M.E., Zakaria M.Z., Aiman A.F. (2019). Optimization of surface roughness in FDM 3D printer using response surface methodology, particle swarm optimization, and symbiotic organism search algorithms. Int. J. Adv. Manuf..

[22] Camposeco-Negrete C. (2020). Optimization of FDM parameters for improving part quality, productivity and sustainability of the process using Taguchi methodology and desirability approach. Prog. Addit. Manuf..

[23] Ramesh M., Panneerselvam K. (2020). Mechanical investigation and optimization of parameter selection for Nylon material processed by FDM. Mater. Today: Proc..

[24] Patel G.C.M., Krishna P., Parappagoudar M.B. (2016). Squeeze casting process modeling by a conventional statistical regression analysis approach. Appl. Math. Model..

[25] Chate G.R., Patel G.C.M., Bhushan S.B., Parappagoudar M.B., Deshpande A.S. (2019). Comprehensive modelling, analysis and optimization of furan resin-based moulding sand system with sawdust as an additive. J. Braz. Soc. Mech. Sci. Eng..

[26] Ganjigatti J.P., Pratihar D.K., RoyChoudhury A. (2008). Modeling of the MIG welding process using statistical approaches. Int. J. Adv. Manuf..

[27] Torres J., Cotelo J., Karl J., Gordon A.P. (2015). Mechanical property optimization of FDM PLA in shear with multiple objectives. JOM.

[28] Mohamed O.A., Masood S.H., Bhowmik J.L. (2016). Mathematical modeling and FDM process parameters optimization using response surface methodology based on Q-optimal design. Appl. Math. Model..

[29] Kumar R., Singh R., Farina I. (2018). On the 3D printing of recycled ABS, PLA and HIPS thermoplastics for structural applications. PSU Res. Rev..

[30] Bachtiar D., Sapuan S.M., Khalina A., Zainudin E.S., Dahlan K.Z.M. (2012). Flexural and impact properties of chemically treated sugar palm fiber reinforced high impact polystyrene composites. Fibers Polym..

[31] Wang R., Meyer C. (2012). Performance of cement mortar made with recycled high impact polystyrene. Cem. Concr. Compos..

[32] Sathishkumar N., Arunkumar N., Balamurugan L., Sabarish L., Joseph A.S.S. (2020). Investigation of mechanical behaviour and surface roughness properties on copper electroplated FDM high impact polystyrene parts. Advances in Additive Manufacturing and Joining.

[33] Coogan T.J., Kazmer D.O. (2020). Prediction of interlayer strength in material extrusion additive manufacturing. Addit. Manuf..

[34] Chate G.R., Patel G.C.M., Deshpande A.S., Parappagoudar M.B. (2018). Modeling and optimization of furan molding sand system using design of experiments and particle swarm optimization. Proc. Inst. Mech. Eng. E: J. Process Mech. Eng..

[35] Patel G.C.M., Chate G.R., Parappagoudar M.B., Gupta K., Gupta M. (2020). Modelling and optimization of alpha-set sand moulding system using statistical design of experiments and evolutionary algorithms. Optimization of Manufacturing Processes.

[36] Patel G.C.M., Shettigar A.K., Parappagoudar M.B. (2018). A systematic approach to model and optimize wear behaviour of castings produced by squeeze casting process. J. Manuf. Process..

[37] Deshwal S., Kumar A., Chhabra D. (2020). Exercising hybrid statistical tools GA-RSM, GA-ANN and GA-ANFIS to optimize FDM process parameters for tensile strength improvement. CIRP J. Manuf. Sci. Technol..

[38] Patel G.C.M., Lokare D., Chate G.R., Parappagoudar M.B., Nikhil R., Gupta K. (2020). Analysis and optimization of surface quality while machining high strength aluminium alloy. Measurement.

[39] Patel G.C.M. (2021). Experimental modeling and optimization of surface quality and thrust forces in drilling of high-strength Al 7075 alloy: CRITIC and meta-heuristic algorithms. J. Braz. Soc. Mech. Sci. Eng..

[40] Das P., Mhapsekar K., Chowdhury S., Samant R., Anand S. (2017). Selection of build orientation for optimal support structures and minimum part errors in additive manufacturing. Comput. Aided Des. Appl..

[41] Zhang J., Li Y. (2013). A unit sphere discretization and search approach to optimize building direction with minimized volumetric error for rapid prototyping. Int. J. Adv. Manuf..

[42] Srinivasan R., Ruban W., Deepanraj A., Bhuvanesh R., Bhuvanesh T. (2020). Effect on infill density on mechanical properties of PETG part fabricated by fused deposition modelling. Mater. Today Proc..

[43] Rao R.V., Pawar R.B. (2020). Constrained design optimization of selected mechanical system components using Rao algorithms. Appl. Soft Comput..

[44] Rao R.V., Pawar R.B. (2020). Self-adaptive multi-population Rao algorithms for engineering design optimization. Appl. Artif. Intell..

[45] Rao R. (2020). Rao algorithms: Three metaphor-less simple algorithms for solving optimization problems. Int. J. Ind. Eng. Comput..

[46] Pathak V.K., Amit K.S. (2017). Particle Swarm Optimization Approach for Minimizing GD&T Error in Additive Manufactured Parts. Int. J. Manuf. Mater. Mech. Eng..

[47] Rao R. (2016). Jaya: A simple and new optimization algorithm for solving constrained and unconstrained optimization problems. Int. J. Ind. Eng. Comput..

[48] Alsattar H.A., Zaidan A.A., Zaidan B.B. (2020). Novel meta-heuristic bald eagle search optimisation algorithm. Artif. Intell. Rev..

[49] El Nahrawy A.M., Abou Hammad A.B., Shaheen T.I., Mansour A.M. (2020). Sol–gel synthesis and physical characterization of high impact polystyrene nanocomposites based on Fe_2_O_3_ doped with ZnO. Appl. Phys. A..

[50] Zhang Z.C., Li P.L., Chu F.T., Shen G. (2019). Influence of the three-dimensional printing technique and printing layer thickness on model accuracy. J. Orofac. Orthop. Fortschr. Kieferorthop..

[51] Gharbi M., Peyre P., Gorny C., Carin M., Morville S., Le Masson P., Carron D., Fabbro R. (2013). Influence of various process conditions on surface finishes induced by the direct metal deposition laser technique on a Ti–6Al–4V alloy. J. Mater. Process. Technol..

[52] Sabbah A., Romanos G., Delgado-Ruiz R. (2021). Impact of Layer Thickness and Storage Time on the Properties of 3D-Printed Dental Dies. Materials.

[53] Singh J.P., Singh R. (2009). Investigations for statistically controlled rapid casting solution of low brass alloys using three dimensional printing. Int. J. Rapid Manuf..

[54] Santana L., Alves J.L., Netto A.D.C.S. (2017). A study of parametric calibration for low cost 3D printing: Seeking improvement in dimensional quality. Mater Des..

[55] Abeykoon C., Sri-Amphorn P., Fernando A. (2020). Optimization of fused deposition modeling parameters for improved PLA and ABS 3D printed structures. Int. J. Lightweight Mater. Manuf..

[56] Garzon-Hernandez S., Arias A., Garcia-Gonzalez D. (2020). A continuum constitutive model for FDM 3D printed thermoplastics. Compos. B: Eng..

[57] Caminero M.A., Chacón J.M., García-Moreno I., Reverte J.M. (2018). Interlaminar bonding performance of 3D printed continuous fibre reinforced thermoplastic composites using fused deposition modelling. Polym. Test..

[58] Gordeev E.G., Galushko A.S., Ananikov V.P. (2018). Improvement of quality of 3D printed objects by elimination of microscopic structural defects in fused deposition modeling. PLoS ONE.

[59] Dave H.K., Rajpurohit S.R., Patadiya N.H., Dave S.J., Sharma K.S., Thambad S.S., Srinivasan V.P., Sheth K.V. (2019). Compressive strength of PLA based scaffolds: Effect of layer height, infill density and print speed. Int. J. Mod. Manuf. Technol..

[60] Buj-Corral I., Bagheri A., Sivatte-Adroer M. (2021). Effect of Printing Parameters on Dimensional Error, Surface Roughness and Porosity of FFF Printed Parts with Grid Structure. Polymers.

[61] Sörensen K. (2015). Metaheuristics—The metaphor exposed. Int. Trans. Oper. Res..

[62] Angayarkanni S.A., Sivakumar R., Rao Y.R. (2021). Hybrid Grey Wolf: Bald Eagle search optimized support vector regression for traffic flow forecasting. J. Ambient Intell. Humaniz. Comput..

[63] Sibalija T.V., Kumar S., Patel G.M. (2021). A soft computing-based study on WEDM optimization in processing Inconel 625. Neural. Comput. Appl..

[64] Patel G.C.M., Krishna P., Parappagoudar M.B., Vundavilli P.R., Bhushan S.B. (2018). Squeeze casting parameter optimization using swarm intelligence and evolutionary algorithms. Critical Developments and Applications of Swarm Intelligence.

[65] Yuen T.J., Ramli R. (2010). Comparision of Compuational Efficiency Of MOEA\D and NSGA-II for Passive Vehicle Suspension Optimization. ECMS.

